# Consumer Acceptability and Metabolic Health Benefits of Meals Containing Pulses (Chickpeas, Lentils, Peas) for Sedentary Office Workers

**DOI:** 10.3390/foods15183302

**Published:** 2026-09-18

**Authors:** Leandy Bertrand, Keely A. Shaw, Shannon Hood-Niefer, Jongbum Ko, Julianne Gordon, Gordon A. Zello, Philip D. Chilibeck

**Affiliations:** 1 College of Kinesiology, University of Saskatchewan, Saskatoon, SK S7N 5B2, Canada; gaz511@usask.ca (L.B.); jongbum.ko@usask.ca (J.K.); julianne.gordon@usask.ca (J.G.); 2 Faculty of Kinesiology, University of Calgary, Calgary, AB T2N 1N4, Canada; keely.shaw@ucalgary.ca; 3 Saskatoon Food Industry Development Centre, Saskatoon, SK S7M 5V1, Canada; shannonhood@niefer.ca; 4 College of Pharmacy and Nutrition, University of Saskatchewan, Saskatoon, SK S7N 5E5, Canada; gordon.zello@usask.ca

**Keywords:** fibre, lipids, legumes, glucose, insulin

## Abstract

We determined whether improvements in metabolic health occur when sedentary office workers replace their regular workplace lunches with pulse-based meals (containing chickpeas, lentils, or split peas). We first tested 10 meals for favourability in 180 participants randomly given combinations of meal pairs. Based on their rankings, we chose seven meals to include in our clinical study. For the clinical study, we used a randomized, single-blind (researchers blind), cross-over design, where participants (n = 20 females; six males; 39 ± 13 y) were assigned to a pulse-based diet to replace their regular workplace meals or their regular diet for 2 months before crossing over to the other diet. Outcomes included insulin sensitivity, blood lipids, blood pressure, and body composition. Diet phase did not impact insulin sensitivity (Matsuda Index; pulse diet: −5.4 ± 23.5; regular diet: −7.5 ± 21.8; *p* = 0.81). The pulse diet significantly reduced the total cholesterol to high-density lipoprotein cholesterol (HDL-c) ratio (−0.13 ± 0.30 vs. +0.08 ± 0.29; *p* = 0.014), and the low-density lipoprotein cholesterol to HDL-c ratio (−0.17 ± 0.36 vs. +0.03 ± 0.29; *p* = 0.033) compared to the regular diet. There were no significant differences between diet phases for blood pressure or body composition. Pulse-based meals incorporated into the office work environment improved blood lipid ratios, but had no effect on insulin sensitivity. (clinicaltrials.gov identifier: NCT03941704).

## 1. Introduction

Sedentary behaviour, defined as “any waking behaviour characterized by an energy expenditure ≤1.5 METs while in a sitting or reclining posture” [1], is recognized as a serious public health risk for diabetes and cardiovascular disease. As such, occupations that involve long sitting time favour the elevation of risk factors for diabetes and cardiovascular diseases, including body mass index (BMI), blood glucose and insulin levels, lipids (non-high-density lipoprotein; non-HDL), blood pressure, and waist circumference [2,3,4]. Canadian adults (18–64 years) spend about 9.5 h per day engaged in sedentary behaviour [5]. Compared to other occupations, office workers in Canada spend more time engaged in sedentary behavior [6] and therefore may be at increased risk for cardiometabolic diseases associated with long periods of sitting. As a result, office workers represent a group ideal for targeted measures aimed at mitigating the risk factors associated with sedentary behaviour.

Research aimed at reducing sedentary behaviour in the occupational setting and improving metabolic health has been dominated by interventions involving sit-stand and active workstations [7,8,9]. While these interventions align with public health recommendations to reduce sedentary behaviour and are successful in doing so [10], they have limited to no effect on improving metabolic health. For example, a recent review on the effects of sit-stand workstations found that they do not have a clinically significant impact on metabolic biomarkers such as blood pressure, energy expenditure, glucose, triglycerides, insulin, or lipids [7]. Similarly, a recent review investigating the use of treadmill desks in office workplaces found no differences in blood pressure, blood glucose, cholesterol, or body anthropometry despite a significant increase in energy expenditure [11]. On the contrary, interventions involving structured physical activity programs (ex., 1 h exercise session incorporated into the workday) tend to improve anthropometric and biochemical health outcomes [12,13,14]. However, compliance with such interventions tends to be low [12,13], limiting the overall effectiveness.

The impact of nutrition on improving the metabolic risks associated with sedentary behaviour has been largely overlooked. To date, there have been no nutrition-based interventions for alleviating risk factors for diabetes and cardiovascular diseases specifically targeting office workers exposed to long periods of sitting. Pulse-based meals (i.e., meals containing non-oil seed legumes such as chickpeas, lentils, beans, or peas), which are high in fibre and high quality protein and low in glycemic index, are effective for improving body composition, reducing non-HDL lipid levels, and reducing diabetes risk in a variety of populations including older adults [15], individuals with overweight or obesity [16], and healthy young adults on bedrest [17]. These favorable findings along with other evidence supporting the metabolic benefits of pulses [18,19,20,21,22] encouraged us to determine whether improvements in metabolic health can be attained when sedentary office workers replace their regular workplace lunches with pulse-based meals. The primary objective was to determine the effect of incorporating more pulse-based meals on combined blood glucose and insulin response to an oral glucose tolerance test (assessed by the Matsuda Index, a measure of insulin sensitivity [23]) in sedentary office workers. The secondary objective was to determine the effect of a pulse-based diet on glucose and insulin incremental area under the curve, and serum lipids (total cholesterol [TC], low-density lipoproteins [LDL], high density lipoproteins [HDL], and triglycerides). We also determined lipid ratios (TC/HDL ratio, LDL/HDL ratio), body composition (total mass, fat mass, trunk fat mass, percentage fat mass, lean tissue mass), waist circumference, BMI, and blood pressure. Our primary hypothesis was that the pulse-based meals would improve insulin sensitivity (i.e., Matsuda Index). Secondary hypotheses were that the pulse-based meals would improve blood glucose control, lipid concentrations, blood pressure, and body composition in sedentary office workers. A conference abstract on the lipid, glucose, and insulin findings from the study has previously been published [24].

## 2. Materials and Methods

Our study used a randomized single-blind cross-over design to assess the effect of two months of a pulse-based diet versus a control diet on measures of insulin sensitivity, lipids, blood pressure, and body composition in sedentary office workers. As unpleasant taste has been identified as a barrier to consuming more pulses [25,26], and therefore a potential threat to compliance in this study, we conducted a meal favourability study prior to initiation of the main study. We evaluated 10 pulse-based meals and selected the most favourable meals to increase the likelihood that participants in the main study would enjoy the meals and adhere to the pulse-based diet.

### 2.1. Meal Favourability Study

Ten meals were assessed for favourability across 180 participants (126 females, 54 males, 25.9 ± 9.3 y, 71.4 ± 16.4 kg, 171 ± 10 cm, BMI = 24.2 ± 4.2 kg/m^2^). Meals included a raisin chickpea tagine, barley and lentil soup, lentil quinoa salad, rosemary lentil soup, minestrone soup (chickpea-based), vegetable chili (chickpea-based), apricot salad (chickpea-based), chana masala (chickpea-based), autumn salad (chickpea-based), and split pea and barley pilaf. To evaluate the meals, we used a “multi-sample difference test: Balanced Incomplete Block Design Ranking Test” according to Meilgaard et al. [27]. Each participant was given two of the 10 meals (50 g sample/meal) to evaluate. The participants were instructed to taste each of the two meal samples and indicate their preferred meal by giving it a score of 1; the least preferred of the two was given a score of 0. An equal number of all pairs of meal combinations were tested to ensure that all of the 10 meals were tested at the same frequency. Ten meals resulted in 45 possible pairs of meals; each pair was tested four times, twice with one meal presented first, twice with the other meal presented first. Participants were randomly assigned to the meal pairings. Each pair of meals were coded A and B to avoid selection based on name familiarity. Preference ratings were then summed to determine which meals were most preferred. The top seven preferred meals (with one exception—see below) were selected for use in our main cross-over clinical trial. Meal favourability testing was completed in the fall of 2018. This favourability trial was submitted to the University of Saskatchewan Biomedical Research Ethics Board (Ethics number Bio #18–37) and was deemed to be exempt from ethics review (exemption date: 26 February 2018). All participants provided written informed consent prior to participation.

### 2.2. Cross-Over Clinical Trial

#### 2.2.1. Participants

Office workers at the University of Saskatchewan, 18 years and over, whose job involved sitting for more than 5 h per day were eligible for this study. Recruitment occurred from May 2019 to February 2020. Persons who were regular consumers of pulses (i.e., ≥250 g of pulses per week), clinically diagnosed with diabetes, pregnant, taking glucose or lipid-lowering medication were excluded, as well as those who were engaging in ≥20 min of moderate-intensity physical activity per day (i.e., we excluded those who were meeting the physical activity guidelines of ≥150 min moderate-intensity physical activity per week). The eligibility criteria also required that the participants not alter their diet and physical activity during enrollment. Participants were recruited via posters which were displayed throughout campus, advertised on the university’s online bulletin portal and circulated throughout various colleges, workers’ unions (representing office works) and the university’s Wellness Centre. The trial was approved by the University of Saskatchewan Biomedical Research Ethics Board (Ethics number Bio #609, approval date: 2 January 2019) and registered at https://www.clinicaltrials.gov (NCT03941704). People who indicated an interest in the study were screened for eligibility to participate and written informed consent was obtained from eligible participants prior to enrollment.

#### 2.2.2. Experimental Design and Diet Intervention

The study was a randomized, single-blind (researchers blinded), cross-over clinical trial conducted at the University of Saskatchewan. Participants (n = 69) were initially randomized using a computer-based random numbers generator (https://www.calculatorsoup.com/calculators/statistics/random-number-generator.php) to either a pulse-based diet to replace their regular workplace meals (i.e., on five days per week) or their usual regular diet for two months. After two months on their first diet, participants had a one-month washout period, followed by a cross-over to the other diet for another two months. We previously used this same timing in a similar cross-over pulse-diet intervention in older adults and found this wash-out period was sufficient for returning fasting glucose, insulin and lipids to baseline levels [15]. A researcher who was not involved in data collection performed the randomization using a computer-generated allocation schedule with a block size of four, who then informed the research assistant who oversaw the distribution of the meals allocated for the participants’ diet. Participants were notified of diet allocation by this research assistant via email. The researchers responsible for participant recruitment, outcome assessments, and data analysis were blinded to participants’ diet allocation.

Sample size was estimated based on findings from previous clinical trials where changes in the Matsuda Index or lipids were assessed [15,17]. For the Matsuda Index it was previously shown that during short-term induced sedentary behaviour (i.e., four days of bed rest in healthy individuals), a pulse-based diet resulted in an increase of 1.61 ± 1.49 (indicating increased insulin sensitivity) compared to a calorie and macro-nutrient matched moderate glycemic index diet (containing no pulses), which resulted in a decrease of 0.14 ± 1.10 [17]. For an alpha of 0.05, power of 90%, and assuming a correlation between repeated measures of 0.80, the calculated sample size was n = 6 for a cross-over (repeated measures) design. Considering some of our more important secondary measurements, changes in LDL predicted a sample size requirement between 18 and 25 [15,17] whereas changes in HDL predicted a sample size requirement of 40 [17]. Our target recruitment was originally 100; however, our recruitment was halted due to the COVID-19 pandemic, which prevented our office workers from working in their typical office environments.

**Figure A1:**
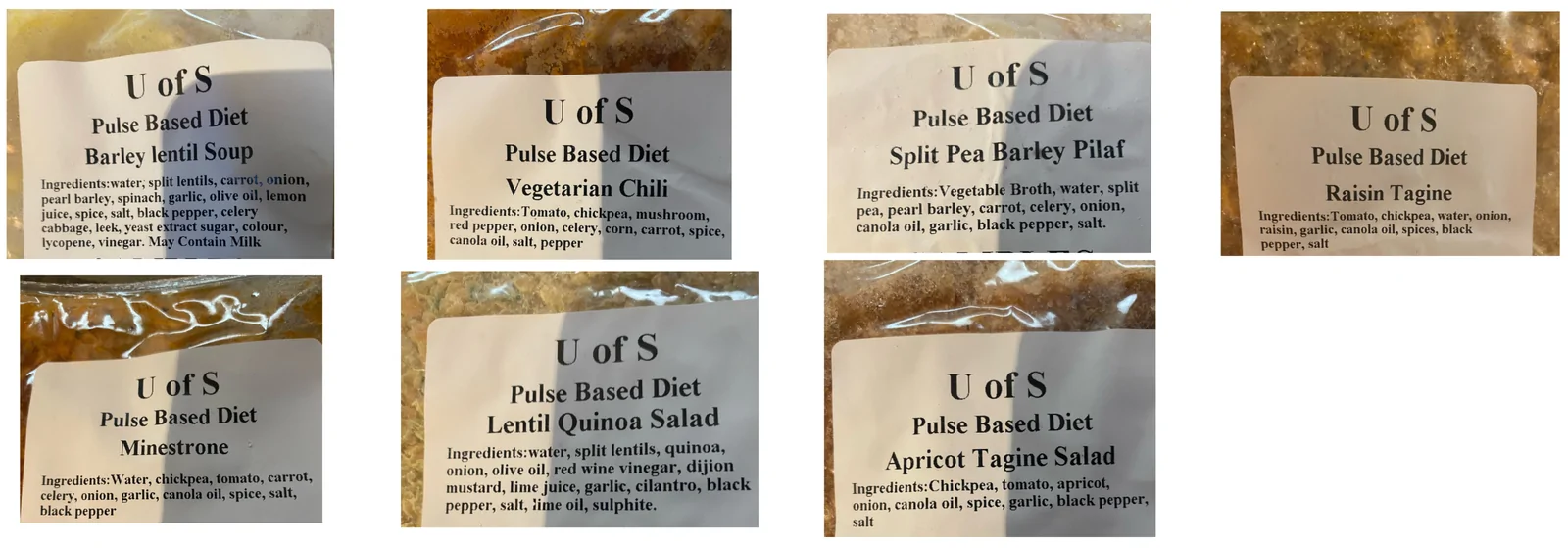
Pictures of the Packages of the Meals Provided to Participants Along with Ingredients Lists.

The pulse-based diet included seven prepacked, ready-prepared meals (derived from the meal favourability study) with chickpeas, lentils, or split peas: minestrone chickpea soup, apricot chickpea salad, vegetable chickpea chili, barley and lentil soup, lentil quinoa salad, raisin chickpea tagine, and split pea and barley pilaf (see Appendix A Figure A1 for pictures of the frozen packaged foods with ingredients). Each meal weighed 450 g and contained approximately 250 g (wet weight) of the featured pulse. The quantity of dietary pulses in the meals is underpinned by evidence from prior investigations which suggests that cardiometabolic benefits are realized at this amount [15,28]. Nutritional information on each meal is provided in Table 1. The meals were stored at −18 °C and distributed to the participants on a weekly basis. Participants on the pulse-based diet were required to replace two meals per day from Monday to Friday with the pulse-based meals provided by the study for two months. More specifically, participants were instructed to replace their workplace lunches and one other daily meal with the pulse-based meals. Participants assigned to the regular diet were asked to continue following their usual diet for two months.

#### 2.2.3. Compliance

Compliance with the pulse-base diet was assessed using weekly logs (total of eight). Each compliance sheet included a list of the seven pulse-based meals, repeated over five weekdays. The following quantities were assigned to each meal: none, 1/4, 1/3, 1/2, 2/3, 3/4, and all. Participants were instructed to select the quantity which corresponded to the amount of each meal eaten daily. For consistency, participants were provided with calibrated bowls to facilitate reheating, consumption, and measurement of meals. Compliance logs were collected during weekly pulse-based meals pick-ups.

#### 2.2.4. Measurements

The primary outcome assessed was the Matsuda Index, determined by the blood glucose and insulin response to an oral glucose tolerance test [23]. The Matsuda Index is calculated as: 10,000/√ (FPG × FPI × G × I), where FPG = fasting plasma glucose, FPI = fasting plasma insulin, G = mean glucose, and I = mean insulin during the two-hour oral glucose tolerance test. Secondary outcomes were glucose and insulin incremental area under the curve, fasting serum lipids (TC, LDL, HDL, triglycerides), body composition (total mass, fat mass, trunk fat mass, percentage fat mass, lean tissue mass), waist circumference, BMI, and blood pressure. We also calculated lipid ratios (TC: HDL and LDL:HDL) from the fasting serum lipid measures. Primary and secondary outcomes were measured before and after each of the two diet phases (i.e., at baseline (week 0), after the first 2-month diet phase, before the second 2-month diet phase, and after the second 2-month diet phase).

##### Blood Collection and Serum Assays

Blood collection occurred in the morning (7 a.m. to 9 a.m.) after a 12-h fast by a trained phlebotomist. On arrival, baseline samples (0 min) of blood were collected in sterile tubes to assess fasting glucose, insulin, and blood lipids. As part of the blood glucose tolerance and insulin tests, additional blood samples were collected 30, 60, 90, and 120 min after consuming 75 g of glucose tolerance test beverage (Thermo Scientific™ NERL™ Trutol™, Pittsburgh, PA, USA). Serum samples were collected from the blood samples after a 30 min period (to allow blood clotting) by centrifugation. Colorimetric assays were performed in-lab to determine the concentrations of glucose (BioAssay Systems™ EnzyChrom™ Glucose Assay Kit, Catalog number EBGL-100, Hayward CA, USA), insulin (ALPCO™ Insulin Enzyme-linked Immunoabsorbant Assays, Catalog number 80-INSHU-eo1, Salem, NH, USA) TC (BioAssay Systems™ EnzyChrom™, Total Cholesterol Assay Kit, Catalog number ECCH-100, detection limit 0.13 mmol/L, linearity up to 7.8 mmol/L, Hayward, CA, USA), LDL, and HDL (BioAssay Systems™ EnzyChrom™ HDL and LDL/VLDL Assay Kit, Catalog number EHDL-100, detection limit 0.13 mmol/L, linearity up to 7.8 mmol/L, Hayward, CA, USA), and triglycerides (BioAssay Systems™ EnzyChrom™ Triglyceride Assay Kit, Catalog number ETGA-200, Linear detection range from 0.01 to 1.0 mmol/L, Hayward, CA, USA). Within-assay % coefficients of variation were <4.0%.

##### Body Composition and Anthropometric Measures

Body composition (total body mass, fat mass, trunk fat mass, % fat mass, lean tissue mass) was determined by Dual Energy X-ray Absorptiometry (QDR Discovery Wi; Hologic Inc., Bedford, MD, USA). The coefficients of variation for these measurements range from 1.0% (whole body lean tissue mass) to 3.0% (whole body fat mass) [29]. Resting blood pressure was measured using an automated blood pressure monitor (BIOS Medical Diagnostic Precision Series 10.0, Newmarket, ON, Canada) after 5 min of comfortable sitting. BMI was determined by dividing participants’ total mass (kg) by height (m^2^). Waist circumference was measured at the superior border of the iliac crest using a measuring tape.

##### Dietary Intake

Dietary intake was measured by repeated 24-h dietary recalls before each diet phase, at the mid-point (i.e., one month), and at the end (i.e., two months) of each diet phase. At each time point, dietary intake was collected using three non-consecutive 24-h dietary recalls (two weekdays and one weekend day). Face-to-face 24 h dietary recall interviews were conducted with participants at the first time-point by a trained nutritionist. During this interview, the process was thoroughly explained to participants, and they were given a copy of the Statistics Canada Food Model Booklet to facilitate future remote dietary recalls. Within the same week, the second and third 24 h dietary recall interviews were conducted via telephone. At midpoint, the three 24 h dietary recall interviews were conducted through telephone interviews only within the same week. At the 2-month follow-up, dietary intake was measured following the same procedures used at the first time point (one in person and two telephone interviews). The collected data were entered into the Food Processor Nutrition Analysis software (ESHA Research, version 11.1; Salem, OR, USA) to compare participants’ dietary intake across both diet phases.

##### Statistical Analysis

Data analysis included only those who completed the full intervention (i.e., complete-case analysis). Differences between those who completed the study and those who dropped out were analyzed using an independent samples *t*-test. Incremental and total areas under the blood glucose and insulin curves (i.e., incremental from baseline) were calculated as described previously [16]. A two-factor (diet × time) repeated measures ANOVA with a least-squared differences post-hoc test on any interactions was used to determine differences between and within diet phases from baseline to two months for most outcome variables. Responses of blood glucose and insulin to the oral glucose tolerance tests were assessed by a three-factor (diet × time during the test [i.e., 0, 30, 60, 90, and 120 min] × time during the diet phase [i.e., baseline, 2-months]) repeated measures ANOVA to determine if the diets resulted in any changes over time at any point in the oral glucose tolerance tests. Sex was initially also included as a factor in our analyses, but after determining there were no sex × diet, sex × time, or sex × diet × time interactions, this was removed to preserve statistical power. We also assessed the effect of order of the intervention by comparing the changes during phase 1 to changes during phase 2 (i.e., irrespective of diet) with a dependent samples *t*-test. Statistical significance was set at *p* < 0.05. Effect sizes were calculated using Glass’ Delta (i.e., dividing the difference for changes between pulse and regular diet phases by the standard deviation of the change score for the regular diet phase). Effect sizes ≤ 0.5 were considered small, >0.5 and <0.8 were considered medium, and ≥0.8 were considered large. For the participants who completed the cross-over study, satisfaction with the meals was evaluated by a 9-point Likert scale with participants scoring the meals on appearance, texture, flavour, and overall liking. This provided a detailed evaluation of longer-term satisfaction with the meals, as opposed to our initial one-time meal favourability study.

## 3. Results

### 3.1. Meal Favourability Study

**Figure 1:**
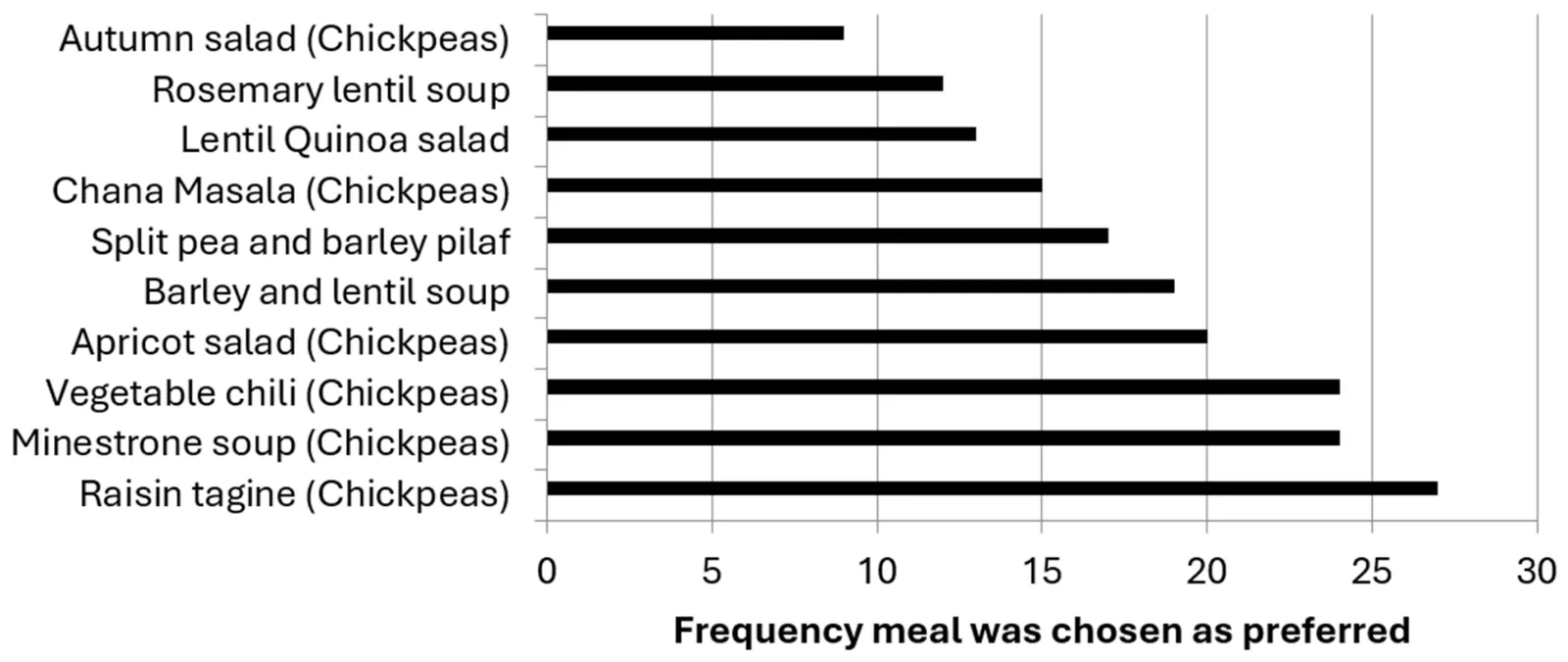
Frequency for which each meal was preferred (n = 180 participants who each evaluated two meals).

The frequency that each meal was chosen as preferred is displayed in Figure 1. The Balanced Incomplete Block Design Ranking Test indicated a significant difference in preference amongst meals (*p* < 0.001). LSD analyses revealed the most preferred meals (raisin tagine, minestrone soup, vegetable chili) were significantly more preferred than chana masala, lentil quinoa salad, rosemary lentil soup, and autumn salad (*p* < 0.05). Autumn salad was also significantly less preferred than barley and lentil soup and apricot salad (*p* < 0.05).

The top six meals were chosen to include in the cross-over study. The lentil quinoa salad (ranked as number eight) was included instead of the chana masala (ranked as number seven) because we wanted to provide a greater variety of pulses (i.e., meals containing chickpeas, lentils, or split peas) and there was no significant difference between the two meals (*p* = 0.64). This resulted in four chickpea-based meals, two lentil-based meals, and one split pea-based meal.

### 3.2. Cross-Over Study

#### 3.2.1. Baseline Characteristics of Participants

**Figure 2:**
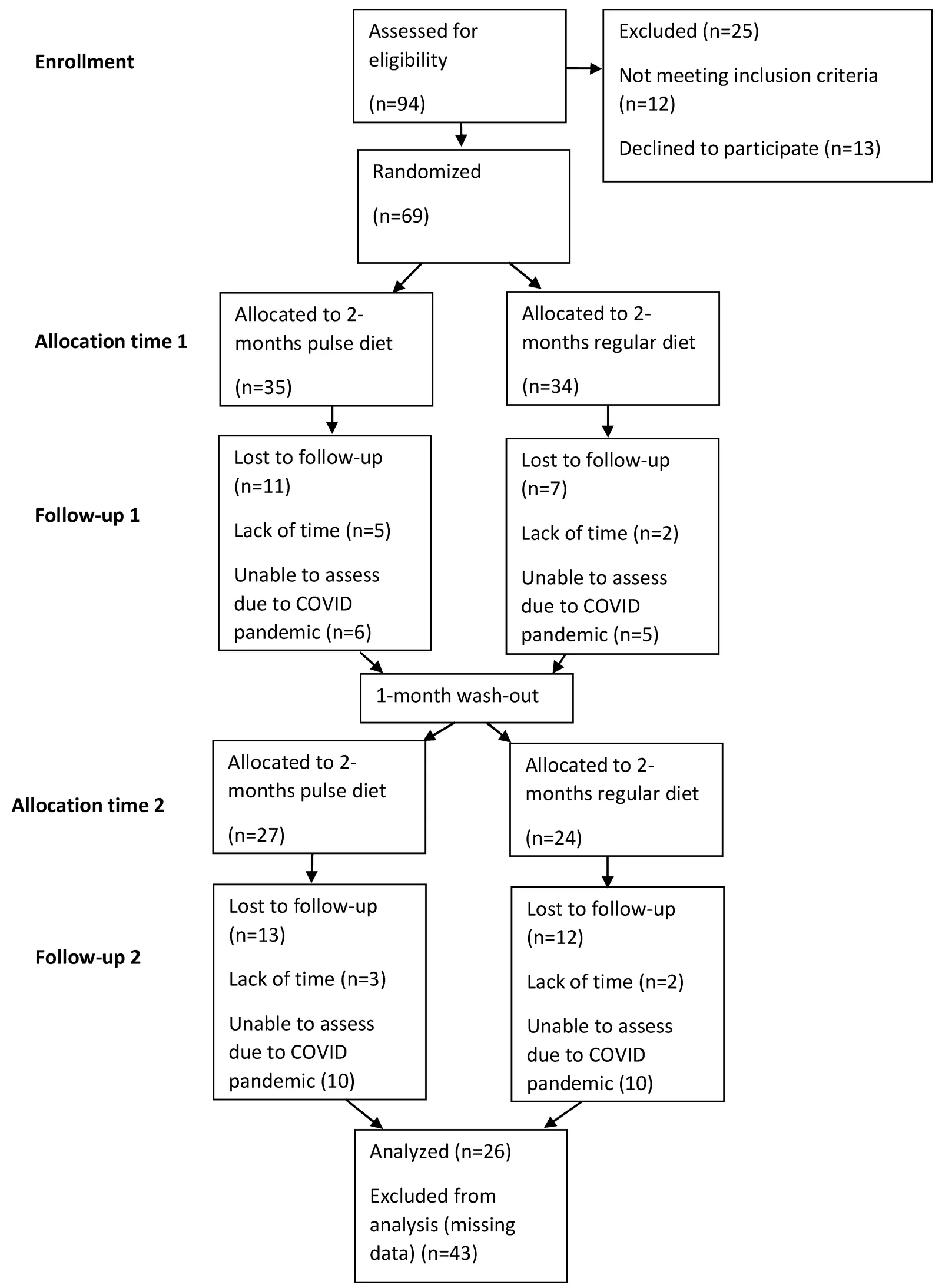
Participant flow through the study.

Participant flow is indicated in Figure 2. Out of the 69 participants who were originally randomized to the intervention, 26 participants (n = 20 women, n = 6 men) completed the study. Twelve participants withdrew because of lack of time and 31 were withdrawn from the study due to the onset of COVID restrictions (i.e., we were no longer permitted to collect data on participants due to COVID lockdowns and workers moving from office to home environments). There were no differences in sedentary time (completers: 591.0 ± 141.4 min/day; dropouts: 634.1 ± 152.5 min/day; *p* = 0.27) or average daily steps (completers: 4317 ± 1878 steps/day; dropouts: 3729 ± 1825 steps/day; *p* = 0.23) between those who completed the research and those who did not. There were no differences in daily step count (pulse diet: 3558 ± 1401 steps/day; regular diet: 4018 ± 1753 steps/day; *p* = 0.45) or sedentary time (pulse diet: 662.1 ± 97.8 min/day; regular diet: 611.3 ± 152.1 min/day; *p* = 0.10) between diet phases.

Baseline characteristics for those randomized first to the pulse-diet compared to those randomized first to the regular diet are presented in Table 2. Based on the sex-specific waist circumference cutoff points (≥94 cm in men and ≥80 cm in women) [30], 65.4% of participants had waist circumference above the cutoff points at baseline. The same percentage of participants (65.4%) were overweight or obese. Systolic and diastolic blood pressure were above recommended levels for cardiovascular disease risk (120 mmHg systolic pressure, 80 mmHg diastolic pressure [30]) in 34.6% and 19.2% of participants respectively. Furthermore, at baseline, 12% of the participants had high triglyceride levels (≥1.69 mmol/L), 54% had low HDL (<1.0 mmol/L men, <1.3 mmol/L women), and 15% high LDL/HDL (>3.0 in men and >2.5 in women) [28]. Fasting serum glucose (<6.9 mmol/L), LDL (<3.36 mmol/L), and TC/HDL (<4.5 in men and <4.0 in women) were optimal for all participants at baseline [30].

#### 3.2.2. Compliance

Results from compliance records indicated that 22 of the 26 participants consumed 100% of the recommended two meals/day during the pulse-based diet phase of the study. The other participants reported consumption of 70–90% of the recommended quantity. There were no serious adverse events reported during the pulse-based diet phase; however, three participants reported episodes of flatulence and two reported bloating after consuming the pulse-based meals. None of these events led to withdrawal from the study.

#### 3.2.3. Effect of Pulse-Based Diet on Outcome Measures

**Figure 3:**
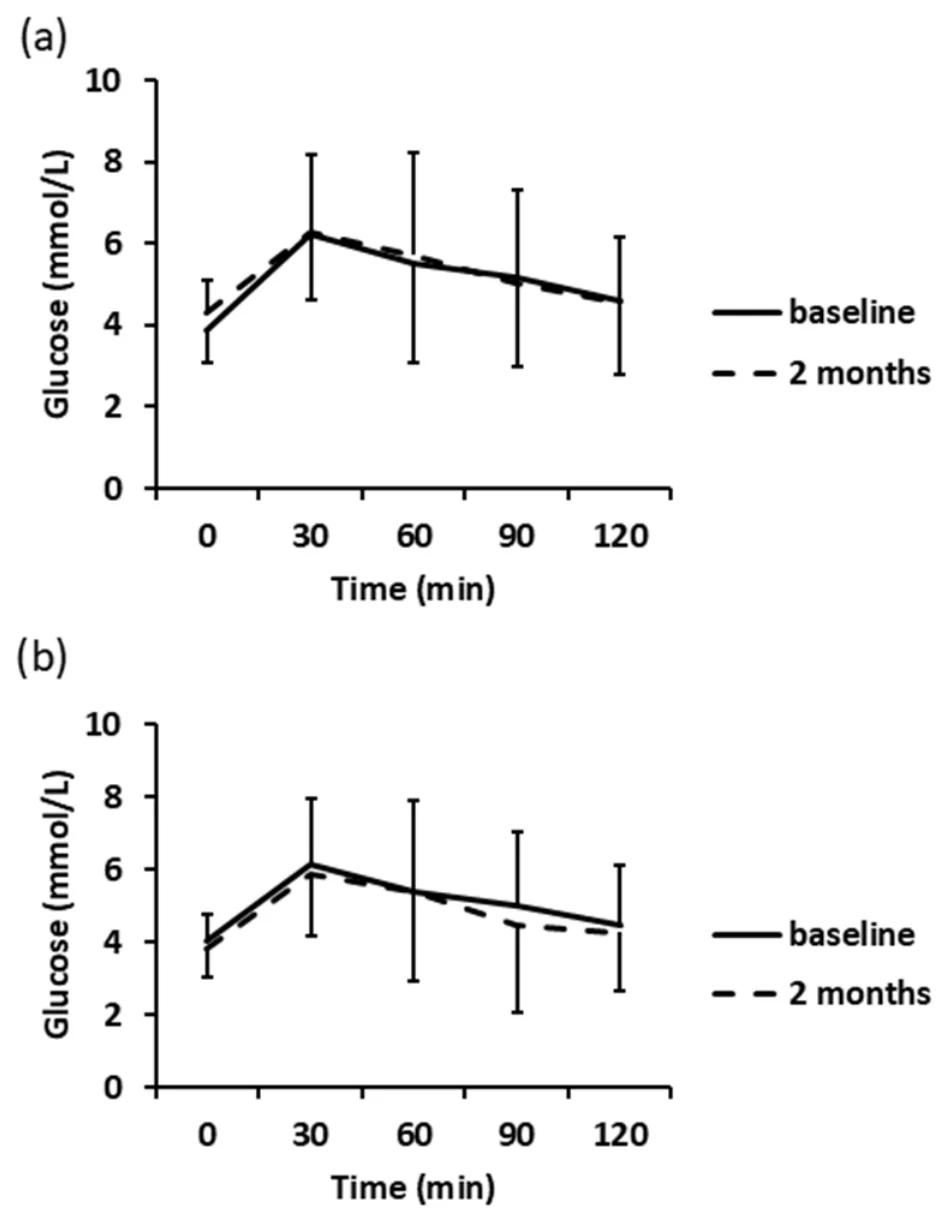

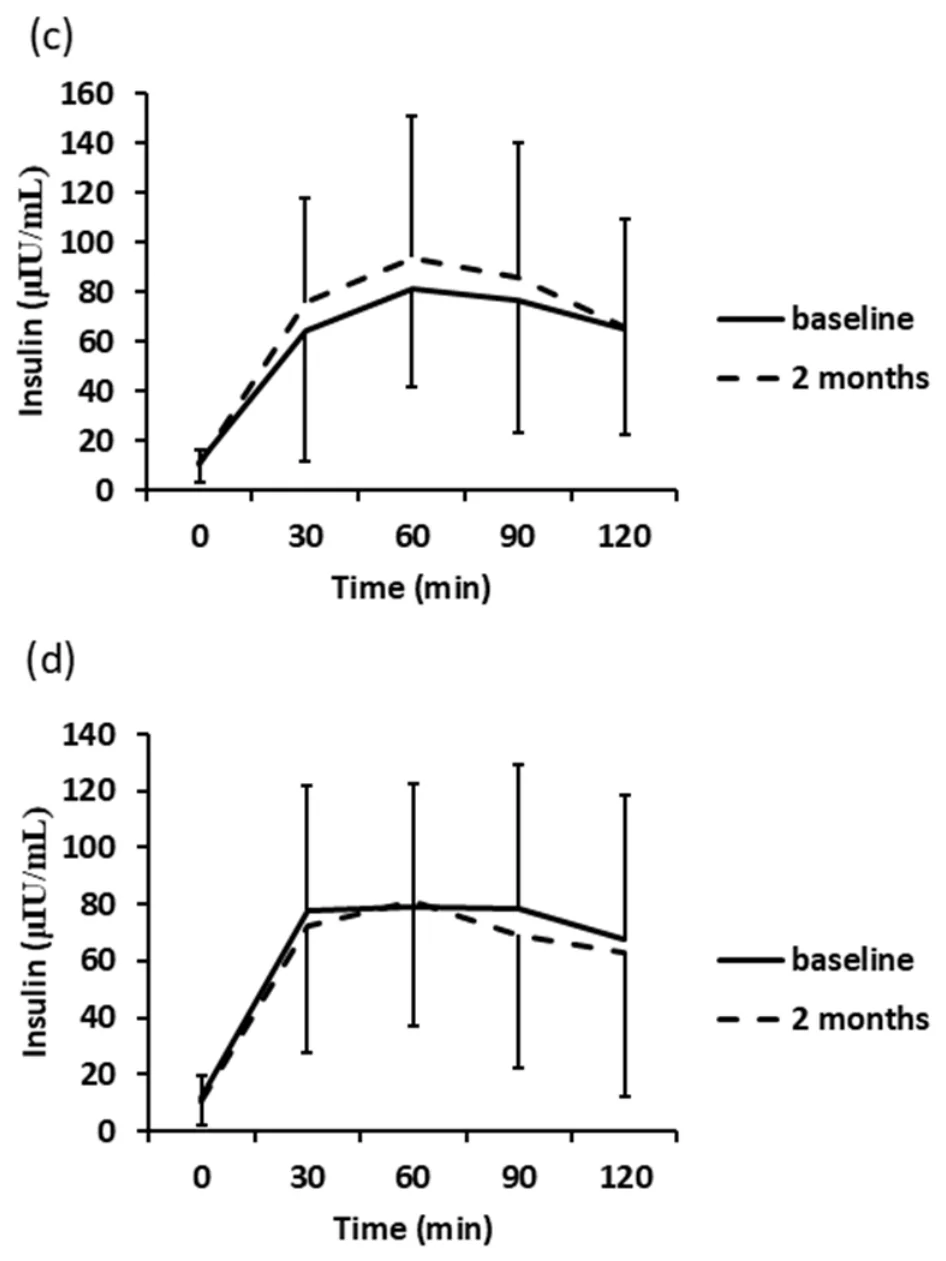
Changes during the pulse-based (**a**,**c**) and regular diet (**b**,**d**) for glucose and insulin during the oral glucose tolerance test from baseline to 2 months. There were no differences from baseline to 2 months and no differences between diet phases for any time point during the oral glucose tolerance tests. Values are means ± SD.

There were no effects of order of diet phase (i.e., phase 1 versus phase 2 irrespective of diet) on any of the outcome variables. Outcome measures at baseline and two months are shown in Table 3. There were no significant differences between the pulse-based and regular diet for changes in the Matsuda Index (Table 3), or any of the glucose or insulin measurements before or during the oral glucose tolerance test from baseline to two months (Table 3; Figure 3). Fasting serum insulin was below detection limit in eight out of 26 participants at baseline; therefore, a sensitivity analysis excluding their data was completed. Removing this data did not alter statistical results. Compared to the regular diet, the pulse-based diet significantly decreased TC/HDL (Table 3; *p* = 0.014), and LDL/HDL (Table 3; *p* = 0.033). There were no diet × time interactions for other outcome measures (Table 3).

#### 3.2.4. Dietary Intake

Table 4 represents the mean daily nutrient and caloric intake at baseline, midpoint (1 month) and end (2 months) of the pulse-based and regular diet. At baseline, there were no significant differences in nutrient and caloric intake between the pulse-based diet and the regular diet. At midpoint, total fibre intake was significantly higher, and dietary cholesterol and sodium intakes were significantly lower in the pulse-based diet. At the end of the pulse-based diet phases, total fibre, folate, magnesium, and zinc intakes were significantly higher, and total fat, cholesterol, vitamin B12, and sodium intakes were significantly lower compared to the end of the regular-diet phase.

#### 3.2.5. Satisfaction with the Meals

**Figure 4:**
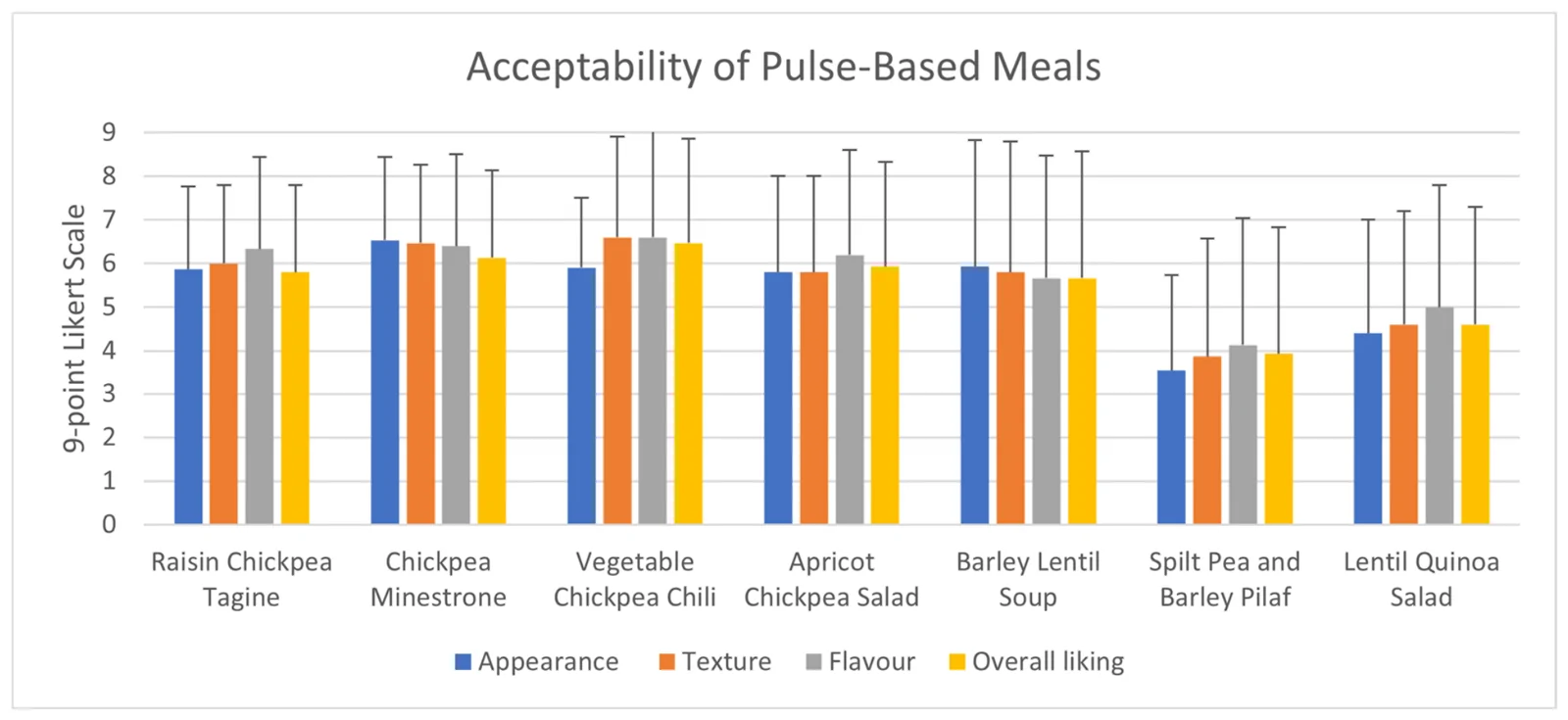
Acceptability of the pulse-based meals for appearance, texture, flavour, and overall liking, based on a 9-point Likert scale where 1 is the worst score possible, and 9 is the best score possible. Values are means ± SD; n = 15.

Figure 4 shows the acceptability of the meals based on appearance, texture, flavour, and overall liking (9-point Likert scale, where 1 is the worst score and 9 is the best score) for participants who completed the cross-over study. This evaluation was added after beginning the intervention, so the first participants to complete the intervention were not captured, leaving n = 15 with meal satisfaction results. The chickpea minestrone and lentil barley soup ranked the best for appearance, and the vegetable chickpea chili and chickpea minestrone soup ranked the best for texture, flavour, and overall liking.

## 4. Discussion

Compared to the regular diet, the pre-packaged pulse-based meals had no effect on insulin sensitivity (i.e., Matsuda Index), our primary outcome measure, but significantly reduced LDL/HDL and TC/HDL with medium effect sizes. In addition to a lack of effect on the Matsuda Index, the pulse-based diet also had no effect on glucose and insulin I-AUC and T-AUC, or insulin or glucose concentrations at any time point before and during the oral glucose tolerance tests. A previous randomized controlled trial (RCT) involving healthy participants subjected to increased sedentary behaviour (i.e., four days of bed rest), found significantly greater changes in the Matsuda Index (indicating increased insulin sensitivity) while on a pulse-based diet (which included meals very similar to the current study) compared to a moderate glycemic index diet that did not contain pulses [17]. The Matsuda Index measured in the study by Gao et al. [17] was much less variable, likely because the participants were in a more controlled environment (i.e., they were all subjected to the same strict bed rest and the diets were more closely monitored; i.e., all food was provided during the bed rest duration). Also, all meals during the pulse-based diet phase in the Gao et al. [17] study contained pulses, whereas in the current study only two meals per day for five days of the week contained pulses. Our results also contrast with a previous study in women with polycystic ovary syndrome, where a pulse-based diet was effective for reducing total area under the curve for insulin during a glucose-tolerance test [28]. This latter study included similar meals as the current study but had a pulse-based intervention that was longer (i.e., 16 weeks) than the current study (~8 weeks) and involved participants who have higher-than-normal insulin responses to glucose. Findings from other studies suggest that it is not uncommon to observe inconsistencies in the glucose-lowering effect of pulses when baseline glucose levels are optimal [18,31] or when there are no significant differences in caloric and macronutrient intake between diets [31,32]. Our study involved normo-glycemic persons; furthermore, there were no statistically significant differences in caloric, carbohydrate, and protein intake between diets.

LDL/HDL and TC/HDL ratios are better predictors of cardiovascular disease than the isolated parameters [33,34,35]. The observed reduction of these ratios in our study suggests that a pulse-based diet can be a useful approach to reduce cardiovascular disease risk among sedentary office workers. The mechanism by which pulses lower these ratios is not clear. It has been suggested that the soluble fibre content of pulses binds to intestinal bile acid, thus inhibiting reabsorption. This stimulates increased absorption of LDL from the blood into the liver as well as transportation of cholesterol (by HDL) from extrahepatic tissues to liver for excretion [22,36]. Fibre from pulses can also be fermented in the colon to form short-chain fatty-acids, which may inhibit hepatic cholesterol synthesis [37]. As with previous pulse-based interventions in older adults [15] or women with polycystic ovary syndrome [28] that showed a significant lipid-lowering effect of a pulse-based diet, the pulse-based diet used in our study resulted in a significantly greater intake of dietary fibre (Table 4). Our study is the first to show a lipid-lowering effect of a pulse-based diet when implemented in an occupational setting where sedentary behaviour is high. While the appropriate dose of pulses to induce lipid-lowering benefits is unclear, interventions delivering about 70% of the dose used in the current study (i.e., delivering 16 g fibre compared to delivering 22 g fibre per day in our study) failed to affect lipid levels [38] whereas studies using pulses delivering a similar dose of fibre as the current study were effective for improving lipids [39].

No statistically significant differences were apparent between the pulse-based diet and the regular diet for changes in systolic and diastolic blood pressure, body weight, BMI, waist circumference, fat mass, trunk fat mass, percent body fat, and lean tissue mass. The minimal changes in body composition are in agreement with a previous pulse-based intervention in older adults that showed only a small reduction in percent fat [15] and a meta-analysis that showed a non-significant trend for reduction of percent body fat during pulse-based diets (MD = –0.34%, 95% CI: –0.71 to 0.03) [20]. Any small reduction in body fat in these studies may be due to the low fat content of pulse-based meals. Regarding blood pressure, evidence from the literature suggests that increased consumption of pulses has a positive effect on systolic and diastolic blood pressure. For example, in a previous trial a similar pulse-based diet (as used in the current study) significantly decreased diastolic blood pressure during a 4-day bed rest compared to a typical moderate-glycemic index diet that did not contain pulses [17]. This similar pulse-based diet also significantly decreased diastolic blood pressure among women with polycystic ovary syndrome [28]. As mentioned previously, the differences between these studies and the current study are that the previous studies used a more tightly controlled diet with every meal containing pulses [17] or used a longer intervention [28]. A meta-analysis of eight RCTs found that consumption of 162 g of pulses per day lowered systolic blood pressure (MD (Mean difference) = −2.25 mmHg, 95% CI: −4.22 to −0.28 mmHg) while there was no significant effect on diastolic blood pressure [21]. Regarding body weight, our finding was not consistent with a meta-analysis of 21 RCTs which found that an intake of 132 g/d of pulses lowers body weight (MD = –0.34 kg, 95% CI: –0.63 to –0.04 kg) [20]; however, our findings were in line with a meta-analysis that showed increased pulse consumption had no significant effect on waist circumference [20].

The pulse-based diet brought about a number of improvements in dietary intake compared to the regular diet. Not only did fibre intake increase at the end of the pulse-based diet but on average participants met the dietary recommendations for fibre intake of 25–38 g/day only when consuming the pulse-based diet [40]. When limiting animal-based proteins, reductions in dietary intake of vitamin B12 cannot be overlooked. Therefore, replacing regular meals with pulse-based meals can account for the reductions in vitamin B12 (Table 4). The pulse-based meals were low in sodium, and this resulted in significant reductions in sodium intake compared to the regular diet. Other noteworthy dietary improvements that can be attributed to increased intake of pulses include reductions in total fat and cholesterol intake and increased intake of folate, iron, magnesium, and zinc. Similar to findings from our study, higher consumption of pulses was associated with higher intakes of folate, magnesium, and zinc among Canadian adults [41]. While some of the changes in diet over the study (Table 4) were most likely due to provision of the pulse-based meals (e.g., change in fibre intake), other changes were most likely due to replacement of participants’ regular meals (e.g., changes in sodium intake). Overall, the meals received acceptable ratings for appearance, texture, and flavour (Figure 4), with the vegetable chickpea chili and chickpea minestrone soup receiving the best ratings, which is reflective of our larger initial meal favourability study (Figure 1).

There are a number of strengths and limitations associated with this study. The impact of increased pulse consumption on the cardiometabolic health of sedentary people is still largely underexplored; therefore, our study was very novel. This study is the first to investigate the effects of a pulse-based diet among sedentary office workers. Findings of this study are limited to sedentary people. The study recruited office workers with >5 h/day of sitting time and with less than 20 min of moderate-intensity physical activity per day; therefore, our results may not extend to individuals with physical activity and sedentary behaviours beyond those limits. The external validity may also be challenged in contemporary office environments that may offer more hybrid or remote work opportunities. Our study included mostly female participants (n = 20) with fewer males (n = 6); therefore, there was an underrepresentation of males in the study. A limitation is that this did not allow adequate comparison between the sexes; however, females are traditionally underrepresented in research on nutrition or lipid-lowering interventions [42,43]. Compliance with the pulse-based diet was assessed using a self-reported compliance sheet provided by the study; therefore, under- and overreporting was possible. Similarly, under- and overreporting inherent in 24-h dietary recalls may have resulted in under- and overestimation of caloric and nutrient intake. The inclusion of a pre-made pulse-based meal during the workday is a pragmatic approach to investigate the role of pulses in improving health outcomes with high external validity; however, the diets were not matched for energy or macronutrients, therefore we cannot be certain that effects are necessarily attributable to the pulses specifically and may be due to meal substitutions. A large number of our intended participants were excluded due to onset of the COVID-19 pandemic and this resulted in lower statistical power for a number of our assessments. The relatively low number of participants might affect our effect size calculations. Relatedly, we did not control for multiple comparisons so as to preserve statistical power, increasing the risk of type I error; therefore, our lipid results should be considered exploratory. Finally, the changes in lipid ratios (i.e., LDL/HDL and TC/HDL) had medium effect sizes, and there were no significant effects for remaining outcomes. One would have to balance the cost of the meals (i.e., 10 meals per week over two months) against the limited benefits for blood lipids.

## 5. Conclusions

Findings from this study demonstrated that ready-to-eat pre-packaged pulse-based meals did not improve insulin sensitivity (as assessed by the Matsuda Index), but improved LDL/HDL and TC/HDL, two important markers of metabolic and cardiovascular disease risk, and also improved dietary intake. Our pulse-based meals were not effective for improving insulin sensitivity, blood pressure, or body composition. Other interventions such as community gardens or organized gym/sport activities at the university might be other options that could benefit university office workers.

## Figures and Tables

**Table 1:** Nutrient composition of the meals.

	Chickpea Minestrone	Chickpea Chili	Barley Lentil Soup	Apricot Lentil Tagine	Raisin Chickpea Tagine	Lentil Quinoa Salad	Split Pea Barley Pilaf	Mean (SD)
Energy (kcal)	270	270	290	530	370	410	390	361 (94)
Protein (g)	13	13	17	25	17	20	20	18 (4)
Carbohydrate (g)	47	46	51	94	62	62	70	62 (17)
Fat (g)	4.5	6	3.5	9	7	9	3.5	6 (3)
Fibre (g)	10	10	10	17	11	10	10	11 (3)
Vit. A (µg RAE)	2	2	0	2	2	0	0	1 (1)
Vit. B_1_ (mg)	0.23	0.23	0.34	0.23	0.23	0.34	0.38	0.3 (0.1)
Vit. B_2_ (mg)	0.13	0.13	0.15	0.13	0.13	0.15	0.1	0.1 (0.2)
Vit. B_3_ (mg)	1	1	2	1	1	2	1.8	1.4 (0.5)
Vit. B_6_ (mg)	0.28	0.28	0.35	0.28	0.28	0.35	0.1	0.3 (0.1)
Vit. C (mg)	2.6	2.6	3	2.6	2.6	3	0.8	2.5 (0.8)
Vit. E (mg)	0	0	0.22	0	0	0.22	0.06	0.1 (0.1)
Vit. K (µg)	8	8	3.4	8	8	3.4	10	7 (2.5)
Folate (µg)	340	340	360	340	340	360	130	316 (82)
Ca (mg)	103	99	77	212	123	70	62	107 (51)
Fe (mg)	5	5	6	10	6	7	4	6.1 (2)
Mg (mg)	96	96	72	96	96	72	72	86 (13)
K (mg)	871	1164	580	1474	976	663	745	925 (312)
Na (mg)	160	120	170	140	120	180	190	154 (28)
Se (µg)	7.4	7.4	5.6	7.4	7.4	5.6	1.2	6 (2)
Zn (mg)	3	3	2.5	3	3	2.5	2	2.7 (0.4)

Abbreviations: RAE = retinol equivalents; Vit = vitamin; Ca = calcium; Fe = iron; Mg = magnesium; K = potassium; Na = sodium; Se = selenium; Zn = zinc. Note that the meals contained no cholesterol, vitamin B_12_, or vitamin D. Note that participants were provided two meals per day.

**Table 2:** Baseline characteristics.

	Total	Received Pulse-Based Diet First (n = 12)	Received Regular Diet First (n = 14)
Age	39 ± 13	43 ± 15	35 ± 10
Total mass (kg)	76 ± 15	76 ± 13	75 ± 16
Sedentary time (min/day)	591.0 ± 141.4	669.7 ± 120.1	534.9 ± 131.3
Average steps/day	4318 ± 1878	3991 ± 1976	4551 ± 1844
BMI (kg/m^2^)	28 ± 5	29 ± 5	27 ± 6
WC (cm)	93 ± 11	96 ± 10	91 ± 12
Fat mass (kg)	26 ± 10	28 ± 9	23 ± 10
% fat mass	34 ± 9	37 ± 7	31 ± 9
Trunk fat mass (kg)	12 ± 5	13 ± 5	11 ± 6
Lean tissue mass (kg)	47 ± 9	45 ± 7	48 ± 10
Systolic blood pressure (mmHg)	115 ± 14	114 ± 11	117 ± 17
Diastolic blood pressure (mmHg)	72 ± 7	71 ± 7	73 ± 8
Fasting glucose (mmol/L)	3.7 ± 0.8	3.5 ± 0.7	3.9 ± 0.9
Fasting insulin (µIU/mL)	9.6 ± 7.5	8.5 ± 3.7	10.5 ± 9.7
LDL (mmol/L)	1.9 ± 0.5	2.2 ± 0.5	1.7 ± 0.5
HDL (mmol/L)	1.2 ± 0.3	1.2 ± 0.4	1.2 ± 0.2
TC (mmol/L)	2.9 ± 0.4	3.0 ± 0.4	2.9 ± 0.5
TG (mmol/L)	1.0 ± 0.9	1.0 ± 0.8	1.1 ± 1.1
LDL:HDL	1.7 ± 0.8	2.0 ± 0.8	1.5 ± 0.6
TC:HDL	2.5 ± 0.6	2.7 ± 0.7	2.4 ± 0.5

All values are means and SD; BMI = body mass index, WC = waist circumference; LDL = low density lipoproteins; HDL = high density lipoproteins, TC = total cholesterol; TG = triglycerides; sedentary time does not include sleep time.

**Table 3:** Outcome measures from baseline to two months (n = 26 for all outcomes).

	Pulse Diet		Regular Diet			
	Baseline	2 Months	Baseline	2 Months	Effect Size	Diet × Time *p*-Value
Matsuda Index	36.0 ± 40.7	27.3 ± 35.7	24.7 ± 39.4	21.1 ± 37.4	0.15	0.65
Glucose I-AUC (mmol/L)	188 ± 175	160 ± 156	162 ± 145	156 ± 148	0.17	0.69
Glucose T-AUC (mmol/L)	633 ± 200	643 ± 213	625 ± 205	593 ± 200	0.40	0.35
Insulin I-AUC (µIU/mL)	6019 ± 3009	6651 ± 3951	6248 ± 3352	5981 ± 3200	0.57	0.26
Insulin T-AUC (µIU/mL)	6793 ± 3668	7641 ± 4397	7287 ± 3940	6928 ± 3849	0.57	0.23
LDL (mmol/L)	2.0 ± 0.6	1.9 ± 0.5	1.8 ± 0.5	1.8 ± 0.4	0.21	0.25
HDL (mmol/L)	1.2 ± 0.3	1.3 ± 0.3	1.3 ± 0.3	1.2 ± 0.3	0.48	0.082
TC (mmol/L)	2.9 ± 0.4	2.9 ± 0.5	2.9 ± 0.4	2.9 ± 0.4	0.21	0.47
TG (mmol/L)	1.0 ± 0.7	0.8 ± 0.6	1.0 ± 0.9	0.9 ± 0.9	0.1	0.71
LDL:HDL	1.73 ± 0.77	1.57 ± 0.56 *	1.56 ± 0.75	1.59 ± 0.74	0.72	0.033
TC:HDL	2.49 ± 0.67	2.36 ± 0.59 * ⁋	2.43 ± 0.62	2.51 ± 0.76	0.74	0.014
Body mass (kg)	75.8 ± 15	75.4 ± 15	75.8 ± 15	75.6 ± 15	0.09	0.66
BMI	27.8 ± 5.1	27.7 ± 4.9	27.8 ± 5.2	27.7 ± 5.0	0.11	0.76
WC (cm)	92.4 ± 11	91.7 ± 11	92.4 ± 12	92.2 ± 11	0.16	0.53
Fat mass (kg)	25.6 ± 10	25.3 ± 9.9	25.2 ± 9.8	25.5 ± 9.5	0.39	0.23
% fat mass	33.6 ± 8.5	33.4 ± 8.9	33.3 ± 8.5	33.6 ± 8.2	0.41	0.13
Trunk fat mass (kg)	11.8 ± 5.1	11.7 ± 5.2	11.8 ± 5.2	11.8 ± 4.8	0.18	0.58
Lean tissue mass (kg)	46.7 ± 9.1	46.8 ± 9.1	46.8 ± 9.2	46.6 ± 8.9	0.27	0.32
SBP (mmHg)	114.8 ± 14	115.5 ± 13	115.9 ± 15	116.8 ± 15	0.04	0.89
DBP (mmHg)	71.4 ± 9.1	71.2 ± 8.0	71.3 ± 8	71.6 ± 9.1	0.10	0.78

All values are means and SD. LDL = low density lipoprotein, HDL = high density lipoprotein, TC = total cholesterol, TG = triglyceride, BMI = body mass index, WC = waist circumference, SBP = systolic blood pressure, DBP = diastolic blood pressure. * 2-month value different from baseline (*p* < 0.05). ⁋ Pulse diet different than regular diet at 2 months (*p* < 0.05).

**Table 4:** Nutrient and caloric intake during pulse-based and regular diet at baseline, midpoint (1 month) and 2 months.

	Pulse-Based Diet			Regular Diet		
	Baseline	1 Month	2 Months	Baseline	1 Month	2 Months
Energy (kcal/d)	1856 ± 542	2730 ± 1378	1729 ± 183	1817 ± 996	2125 ± 692	1786 ± 726
Protein (g/d)	130 ± 61	108 ± 39	83 ± 53	69 ± 43	94 ± 42	71 ± 34
Carbohydrate (g/d)	188 ± 58	438 ± 154	291 ± 43	248 ± 142	269 ± 89	244 ± 110
Fat (g/d)	64 ± 25	78 ± 70	32 ± 27 *	64 ± 32	75 ± 33	54 ± 20
Cholesterol (mg/d)	344 ± 224	48 ± 45 *	30 ± 10 *	162 ± 167	346 ± 268	224 ± 157
Total Fibre (g/d)	13 ± 5.7	55 ± 8 *	47 ± 6 *	13 ± 8	16 ± 3	11 ± 4
Vitamin A (µg RAE/d)	304 ± 375	334 ± 113	336 ± 187	218 ± 172	429 ± 204	282 ± 348
Vitamin B_1_ (mg/d)	1.2 ± 1.1	1.7 ± 1.0	1.6 ± 0.7	1.2 ± 1.1	1.4 ± 1.3	1.4 ± 1.3
Vitamin B_2_ (mg/d)	1.4 ± 1.6	1.4 ± 1.2	0.9 ± 0.3	1.2 ± 1.4	1.4 ± 1.3	1.3 ± 1.2
Vitamin B_3_ (mg/d)	35 ± 40	16 ± 3	15 ± 3	21 ± 23	21 ± 14	17 ± 9.6
Vitamin B_6_ (mg/d)	1.7 ± 0.8	2.0 ± 0.5	1.5 ± 0.3	1.7 ± 2.2	2.1 ± 1.5	1.5 ± 1.2
Vitamin B_12_ (µg/d)	4.3 ± 4.3	0.6 ± 0.7	0.5 ± 0.8 *	2.1 ± 2.4	2.9 ± 3.0	1.1 ± 0.9
Vitamin C (mg/d)	123 ± 139	73 ± 33	60 ± 40	47 ± 76	58 ± 38	97 ± 179
Vitamin D (IU/d)	111 ± 92	84 ± 75	92 ± 104	147 ± 118	171 ± 185	94 ± 85
Vitamin E (mg/d)	2.8 ± 2.6	4.7 ± 1.9	3.9 ± 1.0	4.7 ± 5.1	5.7 ± 5.1	2.8 ± 2.4
Vitamin K (µg/d)	39 ± 54	53 ± 17	59 ± 12	19 ± 27	24 ± 21	29 ± 30
Folate (µg/d)	184 ± 163	1148 ± 118	1106 ± 195 *	140 ± 127	277 ± 185	97 ± 55
Calcium (mg/d)	628 ± 333	964 ± 400	718 ± 161	889 ± 702	705 ± 332	516 ± 260
Iron (mg/d)	12 ± 6	40 ± 7 *	25 ± 6 *	14 ± 9	11 ± 6	9.2 ± 7
Magnesium (mg/d)	154 ± 98	307 ± 48	306 ± 58 *	142 ± 139	146 ± 82	105 ± 39
Potassium (mg/d)	2418 ± 1144	4767 ± 733	3984 ± 472	1573 ± 1111	2204 ± 1046	1774 ± 1379
Sodium (mg/d)	3759 ± 1095	1786 ± 1063 *	891 ± 149 *	2810 ± 1198	3401 ± 1188	3917 ± 1870
Selenium (µg/d)	99 ± 74	28 ± 6 *	32 ± 10	37 ± 44	104 ± 80	53 ± 30
Zinc (mg/d)	5.7 ± 5.1	13.1 ± 1.1	11.7 ± 2.2 *	3.2 ± 2.6	4.8 ± 3.9	3.7 ± 2.4

Data presented as mean ± standard deviation; RAE = retinol activity equivalent; * significantly different from regular diet at the same time point (post hoc test on a significant diet condition × time interaction *p* < 0.05). Note that baseline for the pulse diet phase is without the pulse meals added. The 1-month and 2-month time points for the pulse diet phase includes the pulse-based meals.

## Data Availability

The original contributions presented in this study are included in the article/Supplementary Material. Further inquiries can be directed to the corresponding author.

## Supplementary Materials

The following supporting information can be downloaded at: https://www.mdpi.com/article/10.3390/foods15183302/s1, Data File S1: Sedentary Workers on Pulses data file for Foods.xls.

## References

[1] Sedentary Behaviour Research Network (2012). Letter to the editor: Standardized use of the terms “sedentary” and “sedentary behaviours”. Appl. Physiol. Nutr. Metab..

[2] Saunders T.J., McIsaac T., Douillette K., Gaulton N., Hunter S., Rhodes R.E., Prince S.A., Carson V., Chaput J.-P., Chastin S. (2020). Sedentary behaviour and health in adults: An overview of systematic reviews. Appl. Physiol. Nutr. Metab..

[3] Patterson R., McNamara E., Tainio M., de Sá T.H., Smith A.D., Sharp S.J., Edwards P., Woodcock J., Brage S., Wijndaele K. (2018). Sedentary behaviour and risk of all-cause, cardiovascular and cancer mortality, and incident type 2 diabetes: A systematic review and dose response meta-analysis. Eur. J. Epidemiol..

[4] Biswas A., Oh P.I., Faulkner G.E., Bajaj R.R., Silver M.A., Mitchell M.S., Alter D.A. (2015). Sedentary time and its association with risk for disease incidence, mortality, and hospitalization in adults: A systematic review and meta-analysis. Ann. Intern. Med..

[5] Prince S.A., Melvin A., Roberts K.C., Butler G.P., Thompson W. (2020). Sedentary behaviour surveillance in Canada: Trends, challenges and lessons learned. Int. J. Behav. Nutr. Phys. Act..

[6] Prince S.A., Roberts K.C., Reed J.L., Biswas A., Colley R.C., Thompson W. (2020). Daily physical activity and sedentary behaviour across occupational classifications in Canadian adults. Health Rep..

[7] Chambers A.J., Robertson M.M., Baker N.A. (2019). The effect of sit-stand desks on office worker behavioral and health outcomes: A scoping review. Appl. Ergon..

[8] Mantzari E., Galloway C., Wijndaele K., Brage S., Griffin S.J., Marteau T.M. (2018). Impact of sit-stand desks at work on energy expenditure, sitting time and cardio-metabolic risk factors: Multiphase feasibility study with randomised controlled component. Prev. Med. Rep..

[9] Zhu W., Gutierrez M., Toledo M.J., Mullane S., Stella A.P., Diemar R., Buman K.F., Buman M.P. (2018). Long-term effects of sit-stand workstations on workplace sitting: A natural experiment. J. Sci. Med. Sport..

[10] Chu A.H., Ng S.H., Tan C.S., Win A.M., Koh D., Müller-Riemenschneider F. (2016). A systematic review and meta-analysis of workplace intervention strategies to reduce sedentary time in white-collar workers. Obes. Rev..

[11] Oye-Somefun A., Azizi Z., Ardern C.I., Rotondi M.A. (2021). A systematic review and meta-analysis of the effect of treadmill desks on energy expenditure, sitting time and cardiometabolic health in adults. BMC Public Health.

[12] AlRahma A.M., Habib M.A., Masuadi E., Loney T., Boillat T., Shah S.M., Ahmed L.A., Nauman J. (2025). Effects of a workplace exercise intervention on cardiometabolic health: A randomized controlled trial. BMC Public Health.

[13] Dalager T., Justesen J.B., Murray M., Boyle E., Sjøgaard G. (2016). Implementing intelligent physical exercise training at the workplace: Health effects among office workers-a randomized controlled trial. Eur. J. Appl. Physiol..

[14] Karatrantou K., Gerodimos V., Manouras N., Vasilopoulou T., Melissopoulou A., Mesiakaris A., Theodorakis Y. (2020). Health-Promoting Effects of a Concurrent Workplace Training Program in Inactive Office Workers (HealPWorkers): A Randomized Controlled Study. Am. J. Health Promot..

[15] Abeysekara S., Chilibeck P.D., Vatanparast H., Zello G.A. (2012). A pulse-based diet is effective for reducing total and LDL-cholesterol in older adults. Br. J. Nutr..

[16] Kaviani M., Chilibeck P.D., Yee P., Zello G.A. (2016). The effect of consuming low- versus high-glycemic index meals after exercise on postprandial blood lipid response following a next-day high-fat meal. Nutr. Diabetes..

[17] Gao R., Duff W., Chizen D., Zello G.A., Chilibeck P.D. (2019). The Effect of a Low Glycemic Index Pulse-Based Diet on Insulin Sensitivity, Insulin Resistance, Bone Resorption and Cardiovascular Risk Factors during Bed Rest. Nutrients.

[18] Sievenpiper J.L., Kendall C.W., Esfahani A., Wong J.M., Carleton A.J., Jiang H.Y., Bazinet R.P., Vidgen E., Jenkins D.J. (2009). Effect of non-oil-seed pulses on glycaemic control: A systematic review and meta-analysis of randomised controlled experimental trials in people with and without diabetes. Diabetologia.

[19] Viguiliouk E., Blanco Mejia S., Kendall C.W., Sievenpiper J.L. (2017). Can pulses play a role in improving cardiometabolic health? Evidence from systematic reviews and meta-analyses. Ann. N. Y. Acad. Sci..

[20] Kim S.J., de Souza R.J., Choo V.L., Ha V., Cozma A.I., Chiavaroli L., Mirrahimi A., Blanco Mejia S., Di Buono M., Bernstein A.M. (2016). Effects of dietary pulse consumption on body weight: A systematic review and meta-analysis of randomized controlled trials. Am. J. Clin. Nutr..

[21] Jayalath V.H., de Souza R.J., Sievenpiper J.L., Ha V., Chiavaroli L., Mirrahimi A., Di Buono M., Bernstein A.M., Leiter L.A., Kris-Etherton P.M. (2014). Effect of dietary pulses on blood pressure: A systematic review and meta-analysis of controlled feeding trials. Am. J. Hypertens..

[22] Ha V., Sievenpiper J.L., de Souza R.J., Jayalath V.H., Mirrahimi A., Agarwal A., Chiavaroli L., Mejia S.B., Sacks F.M., Di Buono M. (2014). Effect of dietary pulse intake on established therapeutic lipid targets for cardiovascular risk reduction: A systematic review and meta-analysis of randomized controlled trials. Can. Med. Assoc. J..

[23] Matsuda M., DeFronzo R.A. (1999). Insulin sensitivity indices obtained from oral glucose tolerance testing: Comparison with the euglycemic insulin clamp. Diabetes Care.

[24] Bertrand L., Chilibeck P.D., Hood-Niefer S., Zello G.A. (2021). Consumption of Pulse-Based Meals Improves Lipoprotein Ratios Among Sedentary Office Workers: A Randomized Clinical Trial. Curr. Dev. Nutr..

[25] Kuosmanen S., Niva M., Pajari A.M., Korhonen K., Muilu T., Konttinen H. (2023). Barriers associated with pulse and plant-based meat alternative consumption across sociodemographic groups: A Capability, Opportunity, Motivation, Behaviour model approach. Front. Nutr..

[26] Phillips T., Zello G.A., Chilibeck P.D., Vandenberg A. (2015). Perceived Benefits and Barriers Surrounding Lentil Consumption in Families with Young Children. Can. J. Diet. Pract. Res..

[27] Meilgaard M., Civille G.V., Carr B.T. (1999). Attribute Difference Tests: How Does Attribute X Differ Between Samples. Sensory Evaluation Techniques.

[28] Kazemi M., McBreairty L.E., Chizen D.R., Pierson R.A., Chilibeck P.D., Zello G.A. (2018). A Comparison of a Pulse-Based Diet and the Therapeutic Lifestyle Changes Diet in Combination with Exercise and Health Counselling on the Cardio-Metabolic Risk Profile in Women with Polycystic Ovary Syndrome: A Randomized Controlled Trial. Nutrients.

[29] Chilibeck P.D., Vatanparast H., Pierson R., Case A., Olatunbosun O., Whiting S.J., Beck T.J., Pahwa P., Biem H.J. (2013). Effect of exercise training combined with isoflavone supplementation on bone and lipids in postmenopausal women: A randomized clinical trial. J. Bone Miner. Res..

[30] (2002). National Cholesterol Education Program (NCEP) Expert Panel on Detection, Evaluation, and Treatment of High Blood Cholesterol in Adults (Adult Treatment Panel III). Third Report of the National Cholesterol Education Program (NCEP) Expert Panel on Detection, Evaluation, and Treatment of High Blood Cholesterol in Adults (Adult Treatment Panel III) final report. Circulation.

[31] Saraf-Bank S., Esmaillzadeh A., Faghihimani E., Azadbakht L. (2016). Effects of Legume-Enriched Diet on Cardiometabolic Risk Factors among Individuals at Risk for Diabetes: A Crossover Study. J. Am. Coll. Nutr..

[32] Mollard R.C., Luhovyy B.L., Panahi S., Nunez M., Hanley A., Anderson G.H. (2012). Regular consumption of pulses for 8 weeks reduces metabolic syndrome risk factors in overweight and obese adults. Br. J. Nutr..

[33] Tamada M., Makita S., Abiko A., Naganuma Y., Nagai M., Nakamura M. (2010). Low-density lipoprotein cholesterol to high-density lipoprotein cholesterol ratio as a useful marker for early-stage carotid atherosclerosis. Metabolism.

[34] Millán J., Pintó X., Muñoz A., Zúñiga M., Rubiés-Prat J., Pallardo L.F., Masana L., Mangas A., Hernández-Mijares A., González-Santos P. (2009). Lipoprotein ratios: Physiological significance and clinical usefulness in cardiovascular prevention. Vasc. Health Risk Manag..

[35] Lemieux I., Lamarche B., Couillard C., Pascot A., Cantin B., Bergeron J., Dagenais G.R., Després J.-P. (2001). Total cholesterol/HDL cholesterol ratio vs LDL cholesterol/HDL cholesterol ratio as indices of ischemic heart disease risk in men: The Quebec Cardiovascular Study. Arch. Intern. Med..

[36] Ferreira H., Vasconcelos M., Gil A.M., Pinto E. (2021). Benefits of pulse consumption on metabolism and health: A systematic review of randomized controlled trials. Crit. Rev. Food Sci. Nutr..

[37] Kadyan S., Sharma A., Arjmandi B.H., Singh P., Nagpal R. (2022). Prebiotic Potential of Dietary Beans and Pulses and Their Resistant Starch for Aging-Associated Gut and Metabolic Health. Nutrients.

[38] Zhang X., Irajizad E., Hoffman K.L., Fahrmann J.F., Li F., Seo Y.D., Browman G.J., Dennison J.B., Vykoukal J., Luna P.N. (2023). Modulating a prebiotic food source influences inflammation and immune-regulating gut microbes and metabolites: Insights from the BE GONE trial. EBioMedicine.

[39] Zhang Z., Lanza E., Kris-Etherton P.M., Colburn N.H., Bagshaw D., Rovine M.J., Ulbrecht J.S., Bobe G., Chapkin R.S., Hartman T.J. (2010). A high legume low glycemic index diet improves serum lipid profiles in men. Lipids.

[40] Heart and Stroke Canada Fibre and Whole Grains. https://www.heartandstroke.ca/healthy-living/healthy-eating/fibre-and-whole-grains.

[41] Mudryj A.N., Yu N., Hartman T.J., Mitchell D.C., Lawrence F.R., Aukema H.M. (2012). Pulse consumption in Canadian adults influences nutrient intakes. Br. J. Nutr..

[42] Devries M.C., Jakobi J.M. (2021). Importance of considering sex and gender in exercise and nutrition research. Appl. Physiol. Nutr. Metab..

[43] Khan S.U., Khan M.Z., Raghu Subramanian C., Riaz H., Khan M.U., Lone A.N., Khan M.S., Benson E.-M., Alkhouli M., Blaha M.J. (2020). Participation of Women and Older Participants in Randomized Clinical Trials of Lipid-Lowering Therapies: A Systematic Review. J. Am. Med. Assoc. Netw. Open.

